# Ab Initio Study of the Electronic Properties of a Silicene Anode Subjected to Transmutation Doping

**DOI:** 10.3390/ijms24032864

**Published:** 2023-02-02

**Authors:** Alexander Y. Galashev, Alexey S. Vorob’ev

**Affiliations:** 1 Institute of High-Temperature Electrochemistry, Ural Branch, Russian Academy of Sciences, Sofia Kovalevskaya Str. 22, 620990 Yekaterinburg, Russia; 2 Institute of Chemical Engineering, Ural Federal University Named after the First President of Russia B.N. Yeltsin Mira St. 19, 620002 Yekaterinburg, Russia

**Keywords:** band gap, bond length and energy, first-principle calculations, graphite, multilayer substrates, nickel, nitrogen, silicene, spectrum of electronic states, transmutation doping

## Abstract

In the present work, the electronic properties of doped silicene located on graphite and nickel substrates were investigated by first-principles calculations method. The results of this modeling indicate that the use of silicene as an anode material instead of bulk silicon significantly improves the characteristics of the electrode, increasing its resistance to cycling and significantly reducing the volume expansion during lithiation. Doping of silicene with phosphorus, in most cases, increases the electrical conductivity of the anode active material, creating conditions for increasing the rate of battery charging. In addition, moderate doping with phosphorus increases the strength of silicene. The behavior of the electronic properties of doped one- and two-layer silicene on a graphite substrate was studied depending on its number and arrangement of phosphorus atoms. The influence of the degree of doping with silicene/Ni heterostructure on its band gap was investigated. We considered the single adsorption of Li, Na, K, and Mg atoms and the polyatomic adsorption of lithium on free-standing silicene.

## 1. Introduction

One of the most indispensable and promising devices, lithium-ion batteries (LIBs), are widely used in the field of portable electronic devices, telecommunications, electric vehicles, and electrical networks [1,2]. Graphite is the most well-known intercalation anode material. Graphite anodes are stable over a long cycle. The theoretical storage capacity (TSC) of a graphite anode is 372 mAh g^−1^ [3]. The reversibility of the intercalation/deintercalation cycle is strongly influenced by the solid electrolyte interfacial layer (SEI). This passivating layer is formed on the graphite surface from electrolyte decomposition products during the first few cycles [4]. The SEI prevents the further decomposition of the electrolyte. Another intercalation anode, LTO (Li_4_Ti_5_O_12_), has an even lower specific capacity (175 mAh g^−1^). LTO is distinguished by a long service life, thermal stability, and high rate capability [3]. Modern LIBs are still not without drawbacks, such as the moderate storage capacity of electrode materials, low diffusion rate, overheating problems, dendritic growth, capacity fading, high cost of lithium, and aging problems [5].

Energy storage in lithium-ion batteries (LIBs) is based on the introduction of lithium ions into solid electrodes. Among the known materials, silicon has the highest specific capacity of 4200 mAh g^−1^, which is an order of magnitude higher than that of conventional graphite electrodes [6]. During lithiation, LixSi compounds are formed, and bulk silicon undergoes a huge volume expansion (by about 300%). With such an expansion of the volume during the cycles of lithiation/delithiation, massive cracking of the electrodes occurs, with subsequent loss of capacity [7].

The introduction of new two-dimensional materials into the design of LIBs will solve the main problems associated with their low efficiency. These two-dimensional materials are endowed with many unparalleled advantages over their bulk analogues [8]. Recently, new methods and technologies for synthesizing 2D materials have been developed [9].

Important in the search for a new anode material are the abilities of the anode to accommodate densely packed lithium ions and to provide fast diffusion of lithium to increase the charge–discharge rate. Two-dimensional materials, such as transition metal oxides, dichalcogenides (MO_2_ and MS_2_), and BN, have been studied as anode materials [10,11,12]. Li barrier diffusion in pristine MS_2_ (M = Mo, W, V) is 0.22 eV, which can be reduced by prelithiation MS_2_ [13].

Recent DFT calculations showed that the BeB_2_ and MgB_2_B monolayers have a high Li adsorption energy density, high storage capacity (1749.8 and 1750.9 mAh g^–1^, respectively), and low average open circuit voltage (0.333 and 0.697 V, respectively) [14]. These 2D materials have Li storage capacities 4.5 times higher that of graphite.

Using DFT calculations, various two-dimensional materials were investigated for their application in the design of the anode of a metal-ion battery. Due to the fact that lithium raw materials are expensive and limited, it would seem that they can be replaced by other alkali metals such as Na and K, as well as Mg. The contents of sodium, potassium, and magnesium in the earth’s crust are 2.3, 1.5, and 1.9%, respectively; the content of lithium is only 0.00017%. It was shown [15,16] that when using two-dimensional h-AlC as an anode material for Li-, Na-, and Mg-ion batteries, the achievable TSC is 739.61, 397.58, and 1221.75 mAh g^−1^ for Li-, Na-, and Mg-adsorbed h-AlC monolayers, respectively. Additionally, using DFT calculations, it was found that when a silicon carbide sheet for a Ca-ion battery is used as an anode, the maximum theoretical capacity is 507 mAh g^−1^ [17].

Very promising is the potential to use two-dimensional materials, such as graphene and its silicon analogue, silicene, as the anode material of LIBs. Similar to polycyclic aromatic hydrocarbons the isomer with more Si- hexagons is stable in the case of silicene as well [18]. The buckles in the layers of silicene stimulate the formation of bulk through interlayer interaction. The properties of silicene are largely controlled by the support substrate [19]. Therefore, in order to effectively use the remarkable properties of silicene, an appropriate choice of substrate is necessary. Graphene and silicene (especially the former) have high strength, as determined by their Young’s modulus and bulk modulus [20]. It would be quite appropriate to use a combination of such materials for the manufacture of flexible electrodes. The presence of graphene in the composition of a combined anode reduces the specific capacitance of the electrode but increases its mechanical strength.

Silicene has a much higher surface reactivity than graphene, which is a single layer of graphite. As a consequence, even the adsorption of suitable atoms on one side of the silicene or contact with a single layer material can produce a stable structure. The structures of silicene and graphene are similar. Therefore, the combination of these two materials seems natural. When sheets of silicene and graphene are combined, alternating mixing of Si and C atoms is observed. In this case, the π-bonding network is completely destroyed. In general, the combined structure has the hybridization characteristic of silicene. In addition, the bandgap of the combined structure expands (up to 2.52 eV) due to the sublattice asymmetry in its honeycomb lattice, so that the hybrid material becomes a semiconductor [21].

Graphene can be used as a protective layer to prevent oxidative processes on the material surface [22]. Unlike graphene, silicene oxidizes in air, losing its outstanding electrochemical properties, and it cannot be separated from the substrate. However, the problem of silicene oxidation can be solved, for example, by using Al_2_O_3_ protective layers [23]. Al_2_O_3_-based encapsulation can be carried out when silicene is present on any substrate [24]. This achieves an atomically clear and chemically intact Al_2_O_3_/silicene interface.

Two-dimensional phosphorus, such as silicene, is a candidate for safe anodes at high charge rates and high theoretical open-circuit voltage [25]. Due to its low symmetry, phosphorene has a large number of defects [26]. These defects are created much more easily in phosphorene than in graphene or silicene. Defects, as a rule, have little effect on the electronic properties or band gap of phosphorene, but reduce the mechanical strength of this semiconductor.

Increasing the electrical conductivity of the anode material increases the charging and discharging rate of LIB. Doping of silicene with phosphorus (an element of group V) introduces additional uncompensated electrons into the system and, with heavy doping, should significantly increase its electrical conductivity. In addition, it was experimentally established that in phosphorus-doped silicon, the Si–P bond strength is higher than the Si–Si bond strength [27]. It was shown [28] that the strength of graphene increases when it is doped with nitrogen. With the help of nuclear transmutation doping (NTD), in one session of irradiation with thermal neutrons, some of the silicon and carbon atoms are converted into phosphorus and nitrogen atoms, respectively. It is also possible to convert Ni atoms to Cu atoms.

The possibility of obtaining the ^31^P impurity of the stable ^31^P isotope in thin silicon films electrodeposited onto glassy carbon was shown [29]. The concentration of ^31^P atoms reached ∼1.1 × 10^14^ cm^–3^ in thin silicon films after the NTD process. The irradiation of thin ZnO films with thermal neutrons showed that ^64^Zn isotopes were transformed into ^65^Cu isotopes, the existence of which was confirmed by photoluminescence [30]. Thin semiconducting (Ga_1-x_Mn_𝑥_)As p-type films with magnetic properties were obtained from GaAs samples doped by neutron transmutation [31]. The obtained dependence of the magnetization on the magnetic field showed that ferromagnetism existed in the transmuted films. However, it was also found that the composition of the ^10^B_4_C film does not change under the action of slow neutrons within the measurement accuracy [32]. Irradiation did not lead to increase values of residual stresses and lead to deterioration of film adhesion.

The possibility of using silicene as an anode material for LIBs was studied by quantum mechanical calculations based on density functional theory (DFT) approximation [33,34,35,36,37]. Moreover, calculations were carried out for both free-standing silicene and silicene on a graphite and nickel substrate. Sheets of perfect silicene and silicene containing polyvacancies were considered. The present paper predominantly reflects the results of the computational silicene anodes studies, with an emphasis on the effects produced by the transmutation doping of silicene.

## 2. Results

### 2.1. Open-Circuit Voltage of Lithium-Filled Two-Layer Free-Standing Silicene

**Figure 1:**
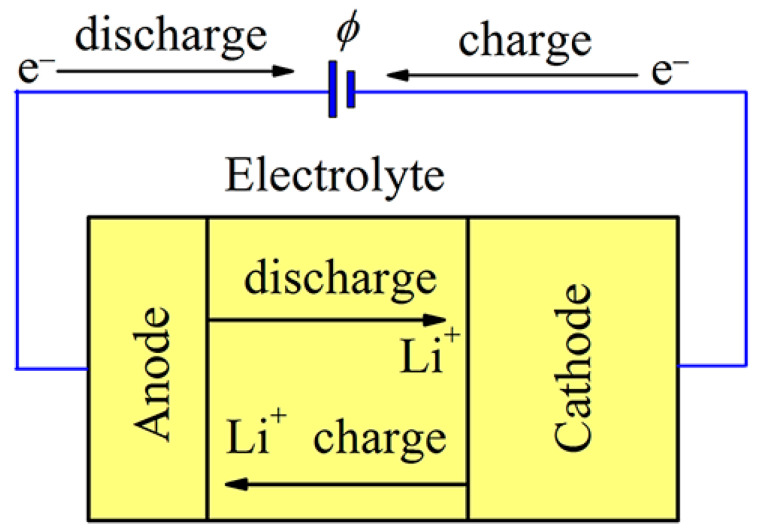
Schematic picture of a rechargeable electrochemical Li cell. When the battery charges, Li is intercalated into the anode; upon discharging, the Li ions are removed from the anode.

A schematic diagram of an electrochemical lithium cell is shown in Figure 1. The cell includes two electrodes, a cathode and an anode, between which there is an electrolyte. The functional purpose of the electrolyte is the transfer of ions between the anode and cathode. The electrolyte has a very small electronic conductivity, which can be neglected. The electrolyte can be either liquid or solid. At present, liquid electrolytes are more often used, which, as a rule, have higher ionic conductivity. The equilibrium voltage difference between the two electrodes is called the open-circuit voltage (OCV). It is desirable for the battery to have a high OCV. The battery’s ability to store charge creates the conditions for high energy density [38]. Thus, to achieve high energy density, it is desirable for the electrode material to have a low molecular weight, and the intercalation voltage should be high.

Cell voltage, capacity, energy, and power capabilities determine the choice of cathode material. A high discharge rate is provided, for example, by a cathode material with a spinel structure (LiMn_2_O_4_). The cathode material usually withstands a large number of lithiation/delithiation cycles. The problem is selecting an anode material that can withstand repeated cycling. The anode material essentially determines how much lithium can be reversibly intercalated, i.e., what determines the capacity of the battery. The most reliable way to find the intercalation energy is to express it from the total energy chemical potential of lithium.

Let *x* = *N*_Li_/*N*_Si_ denote the ratio of the number of Li atoms to the number of Si atoms in the model. Then, without considering the volume and entropy effects, the average voltage of Li*_x_*Si in the range of *x*_1_ ≤ *x* ≤ *x*_2_ can be represented using the expression:

(1) $$\phi =(E_{\mathrm{Li}x1\mathrm{Si}}-E_{\mathrm{Li}x2\mathrm{Si}}+\left(x_{2}-x_{1})E_{\mathrm{Si}}\right)/[(x_{2}-x_{1})q]$$

where $E_{\mathrm{Li}x1\mathrm{Si}}$, $E_{\mathrm{Li}x2\mathrm{Si}}$, and $E_{\mathrm{Si}}$ are the energies of Li*x*_1_Si, Li*x*_2_Si, and metallic Li, respectively; *q* is the charge of ad-atom.

**Figure 2:**
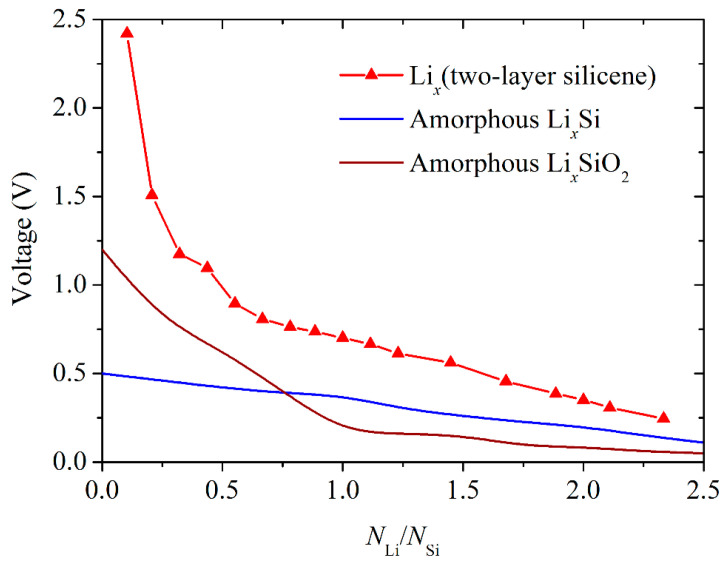
Calculated averaged electrode potential profile for bilayer silicene, amorphous lithium silicide, and lithium silicate.

The voltage profile for the Li*_x_*Si bilayer silicene anode that was investigated by gradually raising the amounts of Li, and the corresponding bulk amorphous silicon and amorphous silicate anode profiles are shown in Figure 2. The profiles for the bulk anodes were determined in DFT calculations [39]. As can be seen from Figure 2, up to *N*_Li_/*N*_Si_ ≈ 0.75, the value ϕ for a bulk silicate anode is higher than the ϕ for a bulk silicon anode. The looser packing in amorphous SiO_2_ is initially more quickly filled with lithium than that in bulk amorphous Si. In this case, the formation of Si–Li and Li–O bonds is more preferable than the preservation of Si–O bonds. This favors the formation of Li*_x_*Si phases. However, at higher values of *N*_Li_/*N*_Si_, the structural rearrangement is inverted, and the ratio in the ϕ values changes in favor of the silicon anode. In the case of a silicene anode, the values of ϕ are significantly higher than those for the above presented bulk material anodes over the entire range of values of the *N*_Li_/*N*_Si_ ratio studied. The calculated average OCV for bilayer silicene (0.81 V) is intermediate between the corresponding OCVs for magnesium batteries, (0.73 V for h-AlC [16] and 0.83 V for phosphorene [40]) and is significantly higher than the average OCV of lithium batteries (0.31 for amorphous Si and 0.35 amorphous SiO_2_) [39].

### 2.2. DFT Simulation of Doped Silicene Anode

#### 2.2.1. Free-Standing Doped Single- and Double-Layer Silicene

**Figure 3:**
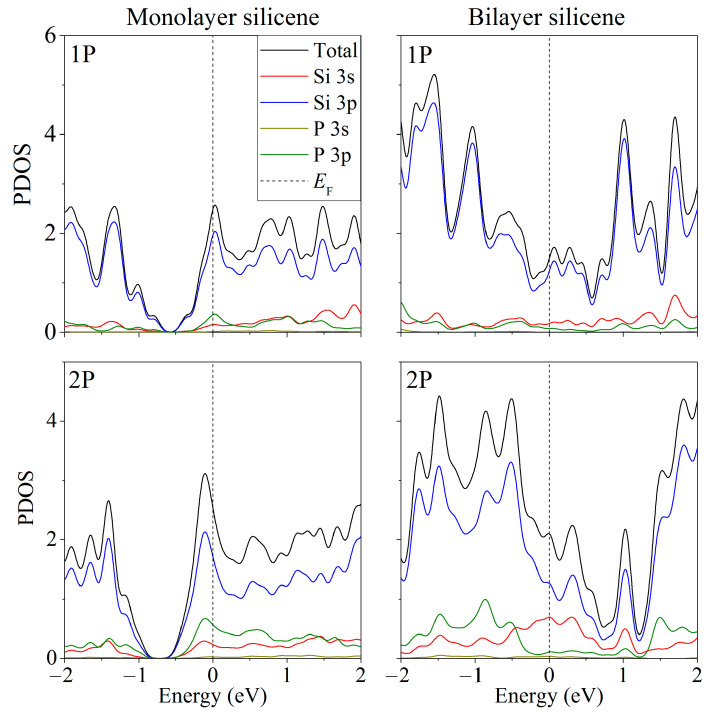
Partial spectra of electronic states of one- and two-layer silicene obtained from the replacement of 1 to 2 silicon atoms by phosphorus.

**Figure 4:**
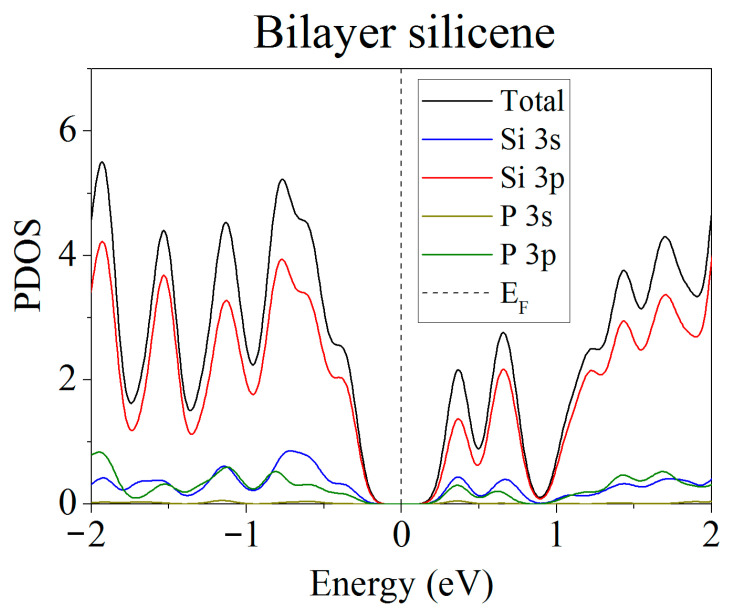
Partial spectrum of electronic states of two-layer silicene obtained after the replacement of 2 Si atoms belonging to each of the lower sublattices by phosphorus.

The partial spectra of electronic states [37] showed that when phosphorus atoms are introduced into silicene instead of Si atoms, the energy gap between the valence band and the conduction band disappears, and silicene acquires conductive properties due to p–p hybridization (Figure 3). However, this does not happen when the substitution of silicene atoms occurs in the lower sublattices of the upper and lower silicene sheets. The PDOS [37] for this case is shown in Figure 4. It can be seen that such a substitution increases the band gap to 0.236 eV.

#### 2.2.2. Doped One- and Two-Layer Silicene on a Graphite Substrate

**Figure 5:**
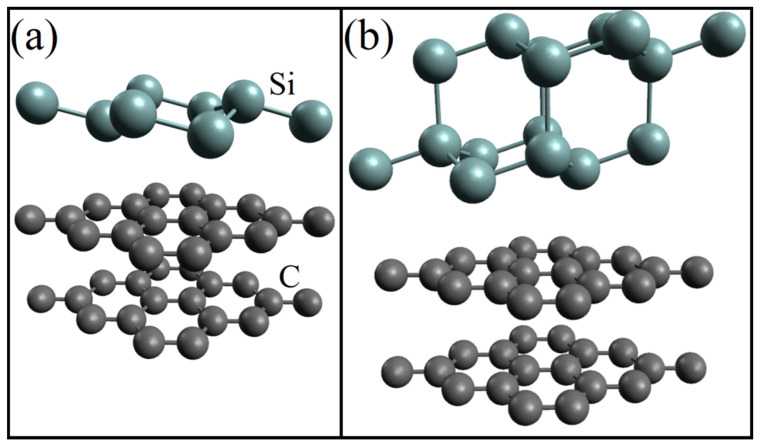
Geometric structure of (**a**) one- and (**b**) two-layer silicene on a graphite substrate after geometric optimization.

After geometric optimization, systems with one- and two-layer silicene on a graphite substrate look as shown in Figure 5 [34]. The optimization causes a significant increase in the distance between the sublattices of only two-layer silicene, while the similar distance in single-layer silicene slightly decreases.

**Figure 6:**
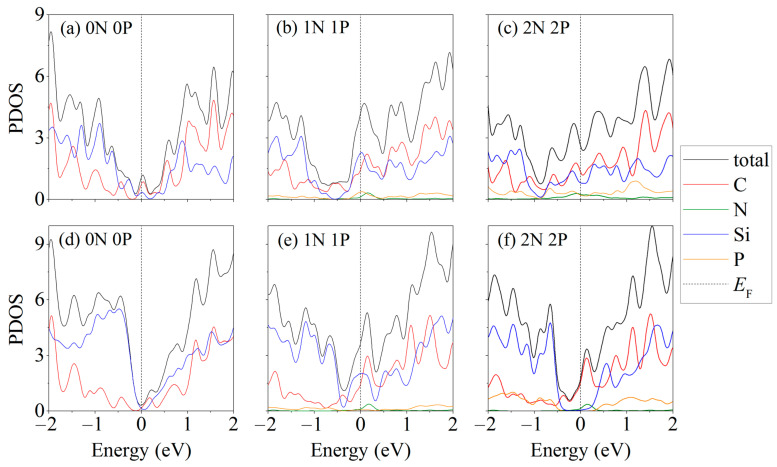
Partial densities of electronic states of doped monolayer (**a**–**c**) and bilayer (**d**–**f**) silicene/graphite substrate systems.

Figure 6 shows the partial densities of electronic states of for some the single-layer (Figure 6a–c) and two-layer (Figure 6d–f) silicene/carbon substrate systems, depending on the nature of the doping [34]. The spectra of the electronic states for systems with a different type of doping are largely similar to those shown in the figure. Most of the systems have conductive properties regardless of the number of phosphorus atoms in the silicene sheet and nitrogen atoms in the carbon substrate. However, the arrangement of P atoms in two-layer silicene on an undoped substrate affects the conductivity of the entire system; so, among the considered systems containing two phosphorus atoms, there may be semiconductors.

**Figure 7:**
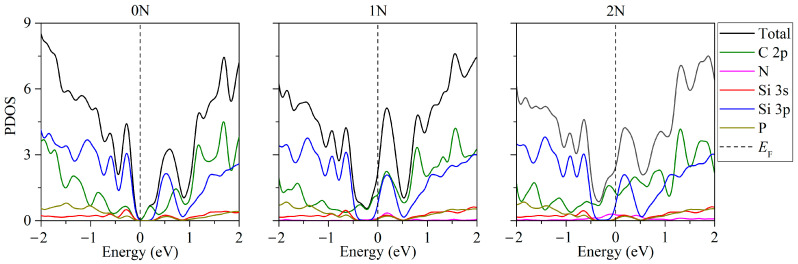
Partial densities of electronic states of doped two-layer silicene/graphite substrate systems in the presence of 2 P atoms in silicene.

Figure 7 shows the partial densities of electronic states of the two-layer silicene-carbon substrate systems in the presence of two phosphorus atoms in silicene and when replacing from zero to two carbon atoms with nitrogen [34]. The replacement of one or two carbon atoms in the substrate with nitrogen atoms leads to a shift in the Fermi level, which results in conductivity over the p orbitals of carbon (Figure 7, 1N, 2N). Nevertheless, the location of two phosphorus atoms in the bottom sheet of silicene in the absence of substituted atoms in the carbon substrate leads to the appearance of semiconductivity with a band gap from 0.009 to 0.023 eV (Figure 7, 0N). In the case of the replacement of Si atoms in the upper sheet of silicene, the system retains its conducting properties.

#### 2.2.3. Lithium Diffusion in Doped Two-Layer Silicene on a Graphite Substrate

Classical MD modeling allowed us to consider larger systems. In particular, this made it possible to observe changes in the shape of silicene sheets between which Li atoms are embedded. Initially, parallel silicene sheets form a flat channel, the walls of which are doped with phosphorus in the following percentage of the number of P atoms with respect to Si atoms: 3%, 6%, 9%, and 18%. P atoms are inserted into preliminarily created mono-, bi-, tri-, and hexavacancies uniformly distributed over the silicene sheets.

The mean square displacement $\langle \Delta r^{2}\rangle$ of Li atoms was calculated in a defect-free silicene channel, as well as in channels with P-doped walls and walls with polyvacancies, in the classical MD model. The values of coefficient *D* are defined through $\langle \Delta r^{2}\rangle$ as

(2) $$D=\frac{1}{6t}\underset{t\to \infty }{\lim}{\left|\langle \Delta r\left(t\right)\right|}^{2}\rangle .$$

**Figure 8:**
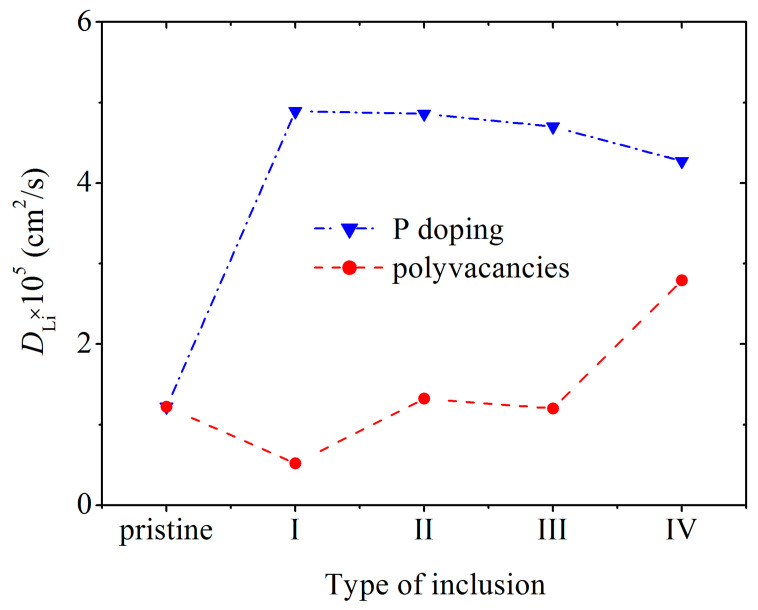
Diffusion coefficient of Li ions during their movement along a pristine silicene channel and a channel whose walls contain P atoms and polyvacancies: I, II, III, and IV—mono-, bi-, tri-, and hexavacancies in silicene, respectively; in all cases, the silicene channel is located on a graphite substrate with a nitrogen content of 5%.

In a doped channel, *D* is much higher than that in channels with walls containing polyvacancies and with pristine walls (Figure 8) [20]. Doping causes a smoothing of the silicene buckles, which enhances the self-diffusion of Li atoms in the channel. The maximum value of *D* is reached at 3% doping with phosphorus. The penetration of P atoms into the channel, the walls of which are more heavily doped, weakens the self-diffusion of Li atoms. The low value of *D* in channels with walls having polyvacancies is associated with a significant deformation of the walls during lithium intercalation. The coefficient *D* increases in the presence of hexavacancies in the channel walls, because Li atoms leave the channel through such holes.

#### 2.2.4. Transmutation Doping of Silicene on a Nickel Substrate

**Figure 9:**
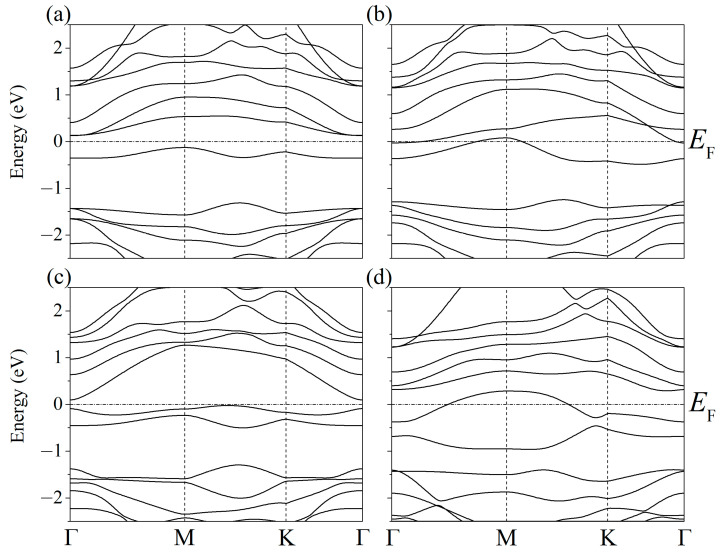
Band structures of silicene modified with one (**a**,**b**) or two (**c**,**d**) phosphorus atoms upon adsorption of one lithium atom in a position above the center of the six-membered ring; (**a**,**b**) P atom is in the lower or upper sublattice of silicene, respectively (**c**) two P atoms are in the lower sublattice or two P atoms are in the upper sublattice; (**d**) one P atom is in the upper sublattice, and the other P atom is in the bottom one.

When one Li atom is adsorbed onto the surface of modified silicene, its band structures take the form shown in Figure 9 [33]. The presence of P atoms in silicene can stimulate the occurrence of a semiconductor–conductor transition. Moreover, the electronic conductivity of silicene depends on its arrangement of P atoms. An indirect band gap Δ = 0.256 eV occurs when there is one P atom (Figure 9a,b). When two P atoms replace Si atoms (Figure 9c,d), the value of Δ is 0.122 eV (P atoms in the lower sublattice) and Δ 0.023 eV (P atoms in the upper sublattice).

Free-standing silicene can be considered a narrow-gap semiconductor (Δ = 0.027 eV) [41]. The partial spectra of the density of electronic states show (Figure 10 in [33]) that the silicene on the Ni substrate acquires conductive properties even in the absence of doping. Conductivity arises due to the interaction of the Ni 3d-electrons with Si 3p electrons.

### 2.3. Alternative Charge Carriers to Lithium

Energy density is the driving force in the search for new electrode materials. The materials used for this should have a large electrochemical capacity and a high available voltage. Active anode materials must not only provide the required capacity, but also have acceptable electronic and ionic conductivity and be stable throughout the entire charging/discharging process. Lithium-ion batteries require materials such as lithium, nickel, and cobalt, which are scarce, expensive, and environmentally damaging. Currently, a search is underway for new charge carriers to create durable and reliable next-generation batteries. Elements such as Na, K, Mg, Ca, and Zn are also capable of acting as charge carriers, similar to lithium ions, but they are much more common and widely available. When using Mg and Zn charge carriers, it is possible to obtain a higher volumetric energy density of the battery than with lithium. The high reactivity of Mg and Ca leads to many chemical complications when creating a battery. The abundance of potassium resources and the low standard potential for its recovery predetermine the use of this element in the electrolyte for batteries. However, cathode materials for potassium batteries have unstable durability, which makes their practical application difficult [42]. Sodium, like lithium, is an alkali metal; Na and Li have similar chemical properties, including ionicity, electronegativity, and electrochemical activity. However, Na^+^ ions are large and have different binding characteristics from Li^+^ ions, which lead to electrochemical behavior that is not reproducible in LIBs [43]. The use of alternative charge carriers to lithium requires new developments in the field of positive and negative electrode materials. This predetermines the importance of studying the physical interaction of silicene with Na, K, and Mg atoms.

#### 2.3.1. Single Adsorption of Li, Na, K, and Mg Atoms on a Free-Standing Silicene Sheet

A check of the stability of the placement of the adsorbed lithium atoms in five selected centers was performed using ab initio molecular dynamics simulation in a Nose–Hoover thermostat. It showed that the most probable location for the placement of Li, Na, K, and Mg atoms is adsorption over the center of a hexagonal silicon ring, while the rest configurations were transformed into the Hollow configuration.

The calculated adsorption energies of Li, Na, Mg, and K atoms on a silicene sheet in a position above the center of the six-membered ring are presented in Table 1. The adsorption energy ($E_{\mathrm{ads}}$) of alkali metals (Li, Na, and K) decreases with increasing atomic number of the element. A certain pattern was found in the values of the energy of the single adsorption of alkali metal atoms on silicene, which can be represented as the following sequence: $E_{\mathrm{ads}}^{\mathrm{Li}}$ > $E_{\mathrm{ads}}^{\mathrm{Na}}$ > $E_{\mathrm{ads}}^{K}$. A certain sequence was also observed for the average bond lengths between alkali metal atoms and silicon atoms: $L_{\mathrm{Si}-K}$ > $L_{\mathrm{Si}-\mathrm{Na}}$ > $L_{\mathrm{Si}-\mathrm{Li}}$. However, the $L_{\mathrm{Si}-\mathrm{Mg}}$ bond length, as well as the adsorption energy of the magnesium ad-atom on the silicene sheet, fall outside of the patterns found for alkali metals.

The band structures and spectra of the electronic states of the systems with adsorbed Li, Na, K, and Mg atoms show that the adsorption of single ad-atoms of lithium, sodium, and potassium in a position above the center of the six-membered ring leads to the metallization of silicene (Figure 11 in [36]). The adsorption of the Mg atom causes an increase in the band gap (up to 0.767 eV) without preserving the Dirac cones.

#### 2.3.2. Polyatomic Adsorption of Lithium on a Free-Standing Silicene Sheet

**Figure 12:**
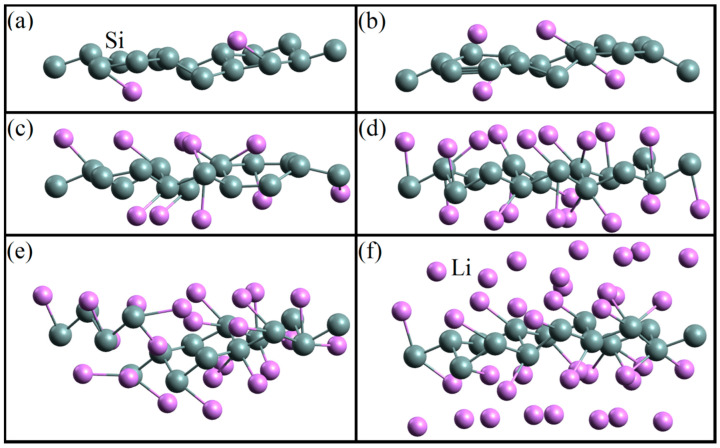
Geometric structure of the silicene/lithium system corresponding to the adsorption ratios: (**a**) 0.125, (**b**) 0.25, (**c**) 0.625, (**d**) 1, (**e**) 1.375, and (**f**) 2.375.

Sequential filling of the silicene sheet with lithium results in the configurations shown in Figure 12 [35]. In this case, the length of Si–Si bonds changes; at *N*_Li_/*N*_Si_ = 1.375, defects are formed due to the incorporation of lithium atoms into the silicene sheet (Figure 12e).

## 3. Discussion

Nuclear transmutation is accomplished through the change of a nucleus to another or multiple other nuclides through a nuclear reaction. The transmutation is usually realized as a result of the interaction of the irradiated material with thermal neutrons. The absorption of fast neutrons prevents the doping of Si. Therefore, a well-thermalized neutron spectrum is used for doping. Natural silicon consists of three isotopes: ^28^Si (abundance: 92.23%), ^29^Si (abundance: 4.67%), and ^30^Si (abundance: 3.10%). The first two types of silicon atoms (^28^Si and ^29^Si), absorbing a thermal neutron, transform into other stable silicon atoms. The capture of thermal neutrons by the ^30^Si isotope creates the conditions for the appearance of the unstable ^31^Si isotope, which undergoes beta decay. The result is a phosphorous atom, ^31^P. The appearance of the P atom in silicon means that it is doped with an n-type element. The transmutation process, during which silicon partially transforms into phosphorus, can be represented by the following reaction:

^30^Si + n → ^30^Si (2.62h) → ^31^P + β^−^. (3)

Here, n denotes a neutron, the letter “h” abbreviates the word “hour”, and β^−^ refers to β radiation.

Element ^31^P is a stable pentavalent impurity. The ^31^P atom has five electrons in its outer shell. Therefore, a type of impurity doping occurs as a result of the above transmutation. Only one nuclear reaction takes place as a result of irradiation of Si with thermal neutrons. The half-life of the ^31^Si element is only 2.62 h. These circumstances make the NTD alloying method appropriate for use on an industrial scale. Large amounts of transmutation-doped silicon can be produced in power nuclear reactors of the RBMK type [44]. Moreover, after neutron irradiation, the high homogeneity of impurities in large ingots of this semiconductor is achieved. The conductivity level of a semiconductor is determined by the amount of dopant introduced into it. The weakly bound electron formed during doping fills the level inside the band gap. It is separated from the conduction band only by a relatively small energy (44 meV for P in Si [45]). At normal temperature, such an electron is excited into the conduction band without the formation of a hole. Doping silicon with phosphorus leads to a situation where electrons are the majority carriers and holes are the minority carriers.

A film anode made of porous silicon ensures stable cycling (for more than 450 cycles) with a specific capacity of 1200 mAh g^−1^ [46]. The present study shows that the efficiency of the silicene/Ni anode can be increased by at least 15% if it is subjected to 3% transmutation doping with phosphorus while doping the nickel part of the anode with copper.

Three main adsorption sites for lithium ions are usually considered. For graphene, these are the hollow site (denote as H) of the C6 hexagon ring, top site (denote as T) of the C atom, and bridge site (denote as B) between two C atoms. Silicene is formed by two sublattices shifted in height. Therefore, in contrast with planar graphene, there are two top sites, T_1_ and T_2_. The adsorption height is defined as the vertical distance from the Li ion to the graphene/silicene center plane. The height barrier for the transition of a lithium atom from one site on the graphene to another is no more than 0.21 Å on average [47]. For silicene, this value reaches 0.27 Å. The energy gain for Li-ion adsorption on the considered surface is defined as the adsorption energy. Therefore, the difference in adsorption energies for different sites determines the energy barrier that an atom must overcome in order to move from one site to another. As for the energy barrier for lithium atom hopping from one site to another, this barrier does not exceed 0.405 eV for graphene and 0.783 eV for silicene.

According to [48], the P-doping of silicene promotes the surface mobility of Li; the diffusion barrier of doped silicene is 0.11 eV versus 0.18 eV for pure silicene. The adsorption energy of lithium on doped silicene decreases by a factor of 3.6 with an increase in the ratio of the number of adsorbed Li atoms from 0.062 to 2.375 [49]. Thus, the behavior of the adsorption energy and the value of the diffusion barrier show the advantage of doped silicenes for use in LIBs compared with pure silicene. An increase in the self-diffusion coefficient of lithium atoms in the phosphorus-doped silicene channel relative to the undoped analogue stimulates an increase in the electrical conductivity and battery charging rates.

The electronic conductivity of the anode material makes it possible to compensate for the intercalation of lithium ions. The low electronic conductivity of the cathode hinders the achievement of high battery performance. A conductive additive can be used to increase the electronic conductivity of the positive electrode. This contributes to faster battery charging. To increase the electronic conductivity, carbon black (CB), which has a high electronic conductivity, is added to the layered cathode material of the Mn_2_O_4_ type [50]. The question still remains open of whether, when calculating the electronic conductivity of a cathode, we should take into account only the CB fraction in it or also take into account the volume fraction of the solid phase, which, in addition to CB, also includes the active material and the binder [51,52].

For the operation of a battery that uses sodium ions as charge carriers, it is necessary to select the appropriate cathode and anode electrodes. While metal oxides and polyanionic compounds can serve as the cathode material in this case [53,54], a reliable and high-performance anode material has not yet been found. The use of a Na/graphite element as an anode makes it possible to achieve a capacity of ~35 mAh g^−1^, which is an order of magnitude lower than the capacity of a Li/graphite element. A possible way to increase the capacitance is to use expanded graphite for making the anode or use a solvent whose molecules are attached to the graphite [55]. Magnesium has a higher melting point than lithium. The energy density can be increased by more than five times when using a magnesium battery instead of a lithium battery [56]. In addition, metallic magnesium does not form dendrites, magnesium is much more abundant in nature than lithium. However, divalent charge carrier ions (such as Zn^2+^, Mg^2+^, or Ca^2+^) have obstacles associated with their migration in solid and organic electrolytes [57]. The use of potassium-ion batteries faces the same difficulties as the use of sodium-ion batteries. The intercalation of potassium into graphite occurs at high temperatures (973 K) when the graphite anode is immersed in KF or KF/AlF_3_ melts. Carbon fibers or “soft” graphite can provide potassium intercalation at lower temperatures [58].

Thus, despite the high cost of lithium, its replacement with other metals in the development of metal-ion batteries still does not seem justified.

Other two-dimensional materials can also be used as the anode material. However, their efficiency is lower than that of silicene. So, thermodynamic calculations show that the stability of free-standing phosphorene intercalated with lithium or sodium is violated when the ratio of the number of Li/P or Na/P atoms reaches 0.5 [59]. One of the reasons for this is the charge transfer from the intercalated alkali metals to the P atoms, as a result of which the interlayer P–P bonds are weakened. In the case of adsorption of lithium on silicene, the limiting value of the Li/Si ratio is twice as large, i.e., equals one [60]. The DFT calculations showed that the achievable Li capacity for graphene/phosphorene/graphene sandwiches is 142–144 mA h/g, and 144, 108, and 96 mA h/g for the monolayer, bilayer, and trilayer free-standing phosphorene without destroying structures, respectively. These values are too low in relation to the corresponding characteristics obtained for free-standing (1384 mA h/g) [61] and two-layer (954 mA h/g) silicene [60].

DFT calculations show that silicene can also be used as anode material for Na-ion batteries. In this case, the calculated capacity for a free-standing silicene is 954 mAh/g, and 730 mAh/g for a graphene-silicene composite [62].

## 4. Materials and Methods

**Figure 13:**
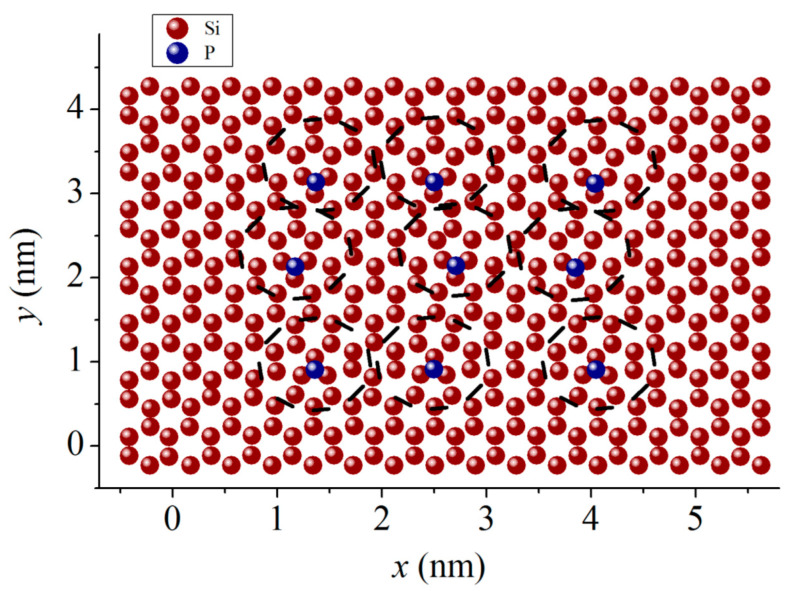
*Xy* projection of the top silicene sheet after filling the doped silicene channel with lithium for 100 ps; dashed circles represent the transformation areas of the P-doped structure.

The structural representation of phosphorus-doped silicene was given [20], where the filling of a silicene channel with a gap of 0.6 nm with lithium was studied. The channel walls were preliminarily doped with phosphorus. The channel was located on a graphite substrate doped with nitrogen. In the central part of the silicene walls of the channel, nine Si atoms were replaced by P atoms approximately evenly over the territory. Regions near P atoms transformed into three neighboring five-membered rings. In Figure 13, P atoms together with the triplets of five-membered rings are marked with dotted circles. Each P atom is bound to three Si atoms. A similar behavior of phosphorus in silicene was observed in quantum mechanical calculations [63]. Such an arrangement of the P atom on silicene corresponds to the maximum binding energy (5.28 eV). If the P atom were located above the center of the hexagonal ring of silicene (hollow position), then the Si–P binding energy would be 2.87 eV.

MD ab initio calculations based on the density functional theory within the general gradient approximation (GGA) and Van der Waals approximation (VDW) for systems containing carbon were performed using the SIESTA 4.0 software package. The calculation of the exchange-correlation functional was based on the Perdue–Burk–Ernzerhof (PBE) [64] and Dion–Rydberg–Schröder–Langreth–Lundqvist (DRSLL) [65] formalism. In the calculations, the Born–Karman periodic boundary conditions were used. The spatial translation period in the z direction in all considered cases was 35 Å.

The integration of the equations of motion in the implementation of ab initio molecular dynamics was carried out by the Verlet method with a time step of 1 fs. Before each MD ab initio calculation, geometric optimization was performed. The dynamic relaxation of atoms continued until the change in the total energy of the system became less than 10^−4^ eV.

The Hamiltonian for the many-body system under consideration includes the kinetic energy operator for electrons, the potential acting on electrons from the nucleus, the electron–electron interaction, the kinetic energy operator for the nucleus, and the internuclear interaction. SIESTA implements strictly localized numerical atomic orbitals. In other words, the Schrödinger equation for an isolated atom is solved based on potential confinement, which makes the orbital equal to zero beyond a given cutoff radius. The use of such an approximation reduces the number of basic functions used, which leads to the very high efficiency of the method, when the memory used and processor time are significantly reduced. A simple physical interpretation (the ability to analyze the population and density of the state) and the achievement of high accuracy are also advantages of this approach. However, choosing a good SIESTA basis and how to extend it (to improve convergence) are not trivial tasks.

There are three important parts of the SIESTA mechanism: pseudopotentials, basis, and k-grid operation.

SIESTA is software based on pseudopotentials, so the GGA pseudo potentials were taken from the SIESTA database. Ultrasoft pseudopotentials were used in the calculations [66]. These pseudopotentials were optimized by minimizing the difference in the results of calculating various electronic configurations obtained in the pseudopotential approximation and taking into account the energy of all electrons.

The assignment of the basis set was performed on the basis of previous data [66]. We used the DZP option, which ensures the cardinality of the basis set (cutoff radius, principal quantum number of the shell, and the angular momentum of the basis orbitals of this shell). In doing so, SIESTA uses internal heuristics to decide which orbitals are actually needed. Using this option involves finding an equilibrium configuration by performing structural relaxation with conjugate gradients for the chosen double basis.

A not-too-dense and therefore not very “expensive” mesh for SIESTA, which nevertheless provided acceptable convergence, was used. The Brillouin zone was determined using 10 × 10 × 1 k-points, which were generated using the Monkhorst–Pack algorithm [67]. The density of the three-dimensional grid used to calculate the electron density was set using the cutoff energy (Meshcutoff) equal to 400 Ry.

Silicene is represented in two ways: single layer, the model of which is defined by a 2 × 2 supercell (eight silicon atoms located in two *xy* planes); and two-layer, defined by two 2 × 2 silicene supercells (sixteen silicon atoms in four *xy* planes). The graphite substrate had two layers. One layer of the graphite substrate was determined by 18 carbon atoms, i.e., by supercell 3 × 3. All atoms of the silicene-carbon substrate system were geometrically optimized. In the model considered here, doping was carried out by replacing silicon atoms with phosphorus atoms, carbon atoms with nitrogen atoms, and nickel atoms with copper atoms.

## 5. Conclusions

Thin doped films of silicon are the most promising anode material for next-generation lithium-ion batteries. Their use in anode design can accommodate the volume expansion during cycling, thus leading to stable battery cycling. In addition, such anode should provide high theoretical specific capacity and safe electrochemical potential.

Existing electrode materials nullify attempts to replace lithium with sodium or another charge carrier (potassium, aluminum). On the whole, the capacitances of the electrodes obtained using sodium ions as charge carriers are, on average, three times lower than for lithium. Replacing Li with Na does not help to overcome the problem of anode volume expansion when it is charged. Therefore, despite the fact that lithium is more expensive than sodium, its use in LIB is advisable to achieve high power and fast battery charging. In addition, the smaller size of lithium ions contributes to higher cycle stability, which is currently more important than obtaining a high energy density.

Ab initio calculations revealed the microstructural changes that occur during lithiation/delithiation and understand the inherent electrochemical mechanisms and design advantages and disadvantages. These data are needed to optimize the performance of LIBs. This study can serve as a practical guide for research related to the development of next-generation LIBs.

## Figures and Tables

**Figure 10:**
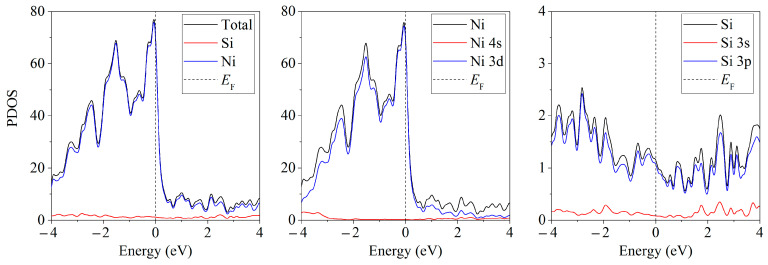
Partial spectra of electronic states of the silicene/nickel substrate system; the substrate contains 4 layers of Ni.

**Figure 11:**
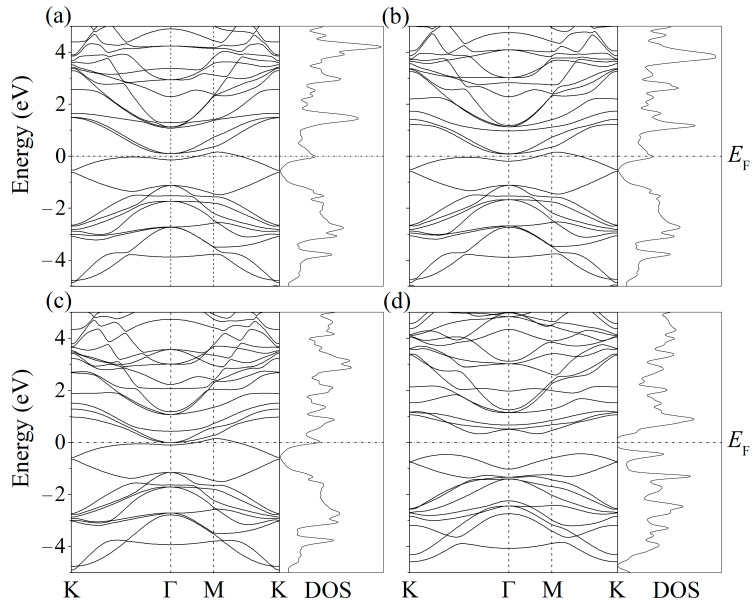
Band structure and density of electronic states (DOS) of silicene after adsorption of a metal atom ((**a**)—Li, (**b**)—Na, (**c**)—K, and (**d**)—Mg) in a position above the center of a six-unit Si ring.

**Table 1:** Bond characteristics of alkali metal and magnesium atoms calculated at their adsorption over the center of a six-member ring of silicene.

Property	Li	Na	K	Mg
$E_{\mathrm{ads}}$, eV	2.082	1.650	1.536	2.667
$E_{b}^{Si-Si}$, eV	4.759	4.761	4.760	4.591
$L_{\mathrm{Si}-\mathrm{Si}}$, Å	2.288	2.285	2.284	2.369
$L_{\mathrm{Si}-\mathrm{Me}}$, Å	2.740	2.990	3.325	2.771

## Data Availability

The data presented in this study are contained within the article.
